# Vaccination Coverage Among Children Aged 19–35 Months — United States, 2016

**DOI:** 10.15585/mmwr.mm6643a3

**Published:** 2017-11-03

**Authors:** Holly A. Hill, Laurie D. Elam-Evans, David Yankey, James A. Singleton, Yoonjae Kang

**Affiliations:** 1 Immunization Services Division, National Center for Immunization and Respiratory Diseases, CDC.

Vaccination is the most effective intervention to reduce morbidity and mortality from vaccine-preventable diseases in young children (*1*). Data from the 2016 National Immunization Survey-Child (NIS-Child) were used to assess coverage with recommended vaccines (*2*) among children aged 19–35 months in the United States. Coverage remained ≥90% for ≥3 doses of poliovirus vaccine (91.9%), ≥1 dose of measles, mumps, and rubella vaccine (MMR) (91.1%), ≥1 dose of varicella vaccine (90.6%), and ≥3 doses of hepatitis B vaccine (HepB) (90.5%). Coverage in 2016 was approximately 1–2 percentage points lower than in 2015 for ≥3 doses of diphtheria and tetanus toxoids and acellular pertussis vaccine (DTaP), ≥3 doses of poliovirus vaccine, the primary *Haemophilus influenzae* type b (Hib) series, ≥3 HepB doses, and ≥3 and ≥4 doses of pneumococcal conjugate vaccine (PCV), with no changes for other vaccines. More direct evaluation of trends by month and year of birth (*3*) found no change in coverage by age 2 years among children included in combined data from the 2015 and 2016 NIS-Child (born January 2012 through January 2015). The observed decreases in annual estimates might result from random differences in vaccination coverage by age 19 months between children sampled in 2016 and those sampled in 2015, among those birth cohorts eligible to be sampled in both survey years. For most vaccines, 2016 coverage was lower among non-Hispanic black* (black) children than among non-Hispanic white (white) children, and for children living below the federal poverty level^†^ compared with those living at or above the poverty level. Vaccination coverage was generally lower among children insured by Medicaid (2.5–12.0 percentage points), and was much lower among uninsured children (12.4–24.9 percentage points), than among children with private insurance. The Vaccines for Children^§^ (VFC) program was designed to increase access to vaccines among children who might not otherwise be vaccinated because of inability to pay. Greater awareness and facilitating use of VFC might be helpful in reducing these disparities. Efforts should also be focused on minimizing breaks in continuity of health insurance and eliminating missed opportunities to vaccinate children during visits to health care providers. Despite the observed disparities and small changes in coverage from 2015, vaccination coverage among children aged 19–35 months remained high and stable in 2016.

The NIS-Child uses a random-digit–dialing sample of landline and cellular telephone numbers to contact parents or guardians of children aged 19–35 months in the 50 states, the District of Columbia, selected local areas, and U.S. territories.^¶^ Parents/guardians are interviewed by telephone to collect sociodemographic and health insurance information for age-eligible children in the household. With consent of parent or guardian, a survey is mailed to all identified vaccination providers to collect dates and types of all vaccines administered to the child. Vaccination coverage estimates use only provider-reported vaccination data. NIS-Child methodology, including weighting procedures, has been described previously.** The household interview response rate^††^ was 33.9% from the combined landline/cell phone sample. Among households with completed interviews, 54.6% had adequate vaccination data from providers,^§§^ yielding 14,988 children available for determination of national coverage estimates for 2016. Logistic regression was used to assess the association of race/ethnicity with vaccination coverage, adjusting for poverty status. T-tests on weighted data were used to evaluate differences in coverage estimates by demographic characteristics; differences were considered to be statistically significant for p-values <0.05. Trends in vaccination coverage by ages 19, 24, and 35 months were evaluated by month and year of birth using weighted linear regression (*3*). Linear trends were estimated using combined data from 2015 and 2016 NIS-Child (births from January 2012 through January 2015), and an expanded analysis of the 2012–2016 data (births from January 2009 through January 2015). Results by age 24 months (2 years) most closely approximate the average age at vaccination assessment in the annual NIS-Child sample (28 months).

## 2016 Vaccination Coverage

Coverage remained ≥90% for ≥3 doses of poliovirus vaccine (91.9%), ≥1 dose MMR (91.1%), ≥1 dose of varicella vaccine (90.6%), and ≥3 doses of HepB (90.5%) (Table 1). Coverage was lowest for ≥2 doses of hepatitis A vaccine (HepA) (60.6%), the combined 7-vaccine series (70.7%),^¶¶^ the HepB birth dose (71.1%), and a completed series of rotavirus vaccine (74.1%). Only 0.8% of children received no vaccinations.

**TABLE 1:** Estimated vaccination coverage among children aged 19–35 months, by selected vaccines and doses — National Immunization Survey-Child, United States, 2012–2016*

Vaccine/Dose	2012	2013	2014	2015	2016
	% (95% CI)	% (95% CI)	% (95% CI)	% (95% CI)	% (95% CI)
**DTaP^†^**					
≥3 doses	94.3 (93.6–95.0)^§^	94.1 (93.2–95.0)	94.7 (94.0–95.4)	95.0 (94.4–95.5)	93.7 (92.8–94.5)^§^
≥4 doses	82.5 (81.3–83.7)^§^	83.1 (81.8–84.3)	84.2 (83.0–85.4)	84.6 (83.5–85.7)	83.4 (82.1–84.6)
**Poliovirus (≥3 doses)**	92.8 (92.0–93.5)^§^	92.7 (91.6–93.6)	93.3 (92.5–94.1)	93.7 (93.0–94.3)	91.9 (90.9–92.9)^§^
**MMR (≥1 dose)^¶^**	90.8 (89.9–91.6)	91.9 (90.9–92.7)	91.5 (90.6–92.4)	91.9 (91.0–92.7)	91.1 (90.1–92.0)
**Hib**					
Primary series**	93.3 (92.5–94.0)	93.7 (92.7–94.5)	93.3 (92.5–94.1)	94.3 (93.7–94.9)	92.8 (91.8–93.6)^§^
Full series**	80.9 (79.7–82.1)	82.0 (80.7–83.3)	82.0 (80.7–83.2)	82.7 (81.5–83.8)	81.8 (80.5–83.0)
**HepB**					
≥3 doses	89.7 (88.8–90.5)^§^	90.8 (89.7–91.7)	91.6 (90.7–92.4)	92.6 (91.9–93.3)	90.5 (89.3–91.5)^§^
Birth dose^††^	71.6 (70.2–73.0)^§^	74.2 (72.8–75.7)^§^	72.4 (70.9–73.9)	72.4 (71.0–73.7)	71.1 (69.5–72.7)
**Varicella (≥1 dose)^¶^**	90.2 (89.4–91.1)	91.2 (90.2–92.1)	91.0 (90.1–91.9)	91.8 (91.0–92.5)	90.6 (89.6–91.5)
**PCV**					
≥3 doses	92.3 (91.5–93.1)^§^	92.4 (91.4–93.3)	92.6 (91.8–93.4)	93.3 (92.5–94.0)	91.8 (90.8–92.7)^§^
≥4 doses	81.9 (80.7–83.0)^§^	82.0 (80.6–83.3)	82.9 (81.6–84.2)	84.1 (83.0–85.2)	81.8 (80.4–83.1)^§^
**HepA**					
≥1 dose	81.5 (80.4–82.6)	83.1 (81.9–84.3)^§^	85.1 (84.0–86.2)^§^	85.8 (84.7–86.8)	86.1 (84.9–87.2)
≥2 doses	53.0 (51.6–54.5)	54.7 (53.1–56.3)	57.5 (55.9–59.1)^§^	59.6 (58.1–61.0)	60.6 (59.1–62.2)
**Rotavirus^§§^**	68.6 (67.2–69.9)	72.6 (71.1–74.0)^§^	71.7 (70.1–73.2)	73.2 (71.8–74.6)	74.1 (72.6–75.5)
**Combined series^¶¶^**	68.4 (66.9–69.7)	70.4 (68.8–71.9)	71.6 (70.2–73.1)	72.2 (70.9–73.6)	70.7 (69.2–72.2)
**No vaccinations**	0.8 (0.6–1.1)	0.7 (0.5–1.1)	0.8 (0.6–1.0)	0.8 (0.6–1.0)	0.8 (0.6–1.0)

**Abbreviations:** CI = confidence interval; DTaP = diphtheria and tetanus toxoids and acellular pertussis vaccine; HepA = hepatitis A vaccine; HepB = hepatitis B vaccine; Hib = *Haemophilus influenzae* type b vaccine; MMR = measles, mumps, and rubella vaccine; PCV = pneumococcal conjugate vaccine.

* For 2012, children born January 2009–May 2011; for 2013, children born January 2010–May 2012; for 2014, children born January 2011–May 2013; for 2015, children born January 2012–May 2014; and for 2016, children born January 2013–May 2015.

^†^ Includes children who might have been vaccinated with diphtheria and tetanus toxoids, or diphtheria and tetanus toxoids and pertussis vaccine.

^§^ Statistically significant (p<0.05) change in coverage compared with previous year.

^¶^ Includes children who may have been vaccinated with measles, mumps, rubella, and varicella vaccine.

** Hib primary series: receipt of ≥2 or ≥3 doses, depending on product type received; full series: primary series and booster dose includes receipt of ≥3 or ≥4 doses, depending on product type received.

^††^ One dose HepB administered from birth through age 3 days.

^§§^ Includes ≥2 doses of Rotarix monovalent rotavirus vaccine (RV1), or ≥3 doses of RotaTeq pentavalent rotavirus vaccine (RV5).

^¶¶^ The combined 7-vaccine series (4:3:1:3*:3:1:4) includes ≥4 doses of DTaP, ≥3 doses of poliovirus vaccine, ≥1 dose of measles-containing vaccine, the full series of Hib (≥3 or ≥4 doses, depending on product type of vaccine), ≥3 doses of hepatitis B vaccine, ≥1 dose of varicella vaccine, and ≥4 doses of PCV.

## Vaccination Coverage by Race/Ethnicity, Poverty Status, and Type of Health Insurance

Compared with white children, black children had lower coverage with ≥3 and ≥4 doses of DTaP, the primary and full series of Hib, ≥3 and ≥4 doses of PCV, ≥2 doses of HepA, the completed rotavirus vaccine series, and the 7-vaccine series (Table 2). For ≥3 doses of DTaP, the primary series of Hib, and ≥3 doses of PCV, these disparities were not statistically significant after adjustment for poverty status; however, for the remaining vaccines, racial/ethnic disparities persisted only among children living at or above poverty (data not shown). For example, coverage with ≥4 doses of DTaP was similar for white and black children below poverty (75.6% and 76.6%, respectively); among children living at or above poverty, coverage rates among white and black children were 86.8% and 77.2%, respectively. Among children at or above poverty, a higher proportion of black children than white children (25.8% of black children compared with 10.4% of white children) were living just above the poverty level (up to 138% of poverty). The proportion of white children living in households with an income to poverty ratio of ≥4 was twice that of black children (41.5% and 20.4%, respectively). Rotavirus vaccination coverage was lower among Hispanic (73.0%) than among white (77.3%) children.

**TABLE 2:** Estimated vaccination coverage among children aged 19–35 months, by selected vaccines and doses, race/ethnicity,* poverty level,^†^ and health insurance status^§^ — National Immunization Survey-Child, United States, 2016^¶^

Vaccine/Dose	Race/Ethnicity							Poverty level		Health insurance status			
	White, non-Hispanic (Referent) (n = 8,794)	Black, non-Hispanic (n = 1,307)	Hispanic (n = 2,727)	American Indian/ Alaska Native, non-Hispanic (n = 214)	Asian, non-Hispanic (n = 731)	Native Hawaiian or other Pacific Islander, non-Hispanic (n = 104)	Multiple races, non-Hispanic (n = 1,111)	At or above poverty (Referent) (n = 11,062)	Below poverty (n = 3,366)	Private only, (Referent) (n = 8,284)	Any Medicaid (n = 5,757)	Other insurance (n = 567)	Uninsured (n = 380)
	% (95% CI)	% (95% CI)	% (95% CI)	% (95% CI)	% (95% CI)	% (95% CI)	% (95% CI)	% (95% CI)	% (95% CI)	% (95% CI)	% (95% CI)	% (95% CI)	% (95% CI)
DTaP**													
≥3 doses	94.6 (93.7–95.3)	90.8 (87.7–93.2)^††^	93.3 (90.6–95.2)	93.6 (87.4–96.9)	96.6 (92.8–98.4)	91.4 (82.6–96.0)	92.8 (88.9–95.4)	94.7 (93.7–95.6)	91.7 (89.8–93.3)^††^	95.9 (95.1–96.7)	92.5 (90.9–93.8)^††^	93.2 (87.0–96.6)	80.2 (72.6–86.2)^††^
≥4 doses	84.8 (83.4–86.2)	76.8 (72.5–80.7)^††^	83.3 (80.0–86.2)	83.5 (75.7–89.2)	86.4 (80.3–90.8)	83.2 (72.2–90.4)	83.6 (79.2–87.2)	85.1 (83.5–86.6)	79.2 (76.7–81.5)^††^	87.3 (85.6–88.9)	81.2 (79.2–83.0)^††^	79.7 (69.9–87.0)	63.2 (54.6–71.0)^††^
**Polio** (≥3 doses)	92.5 (91.3–93.6)	90.3 (87.2–92.7)	91.7 (88.8–93.9)	92.4 (85.8–96.1)	94.7 (90.8–97.0)	91.3 (82.5–95.9)	89.4 (84.2–93.1)	92.5 (91.2–93.7)	90.6 (88.6–92.3)	93.6 (92.2–94.7)	91.1 (89.4–92.9)^††^	92.8 (86.7–96.3)	79.4 (71.8–85.4)^††^
**MMR**^§§^(≥1 dose)	91.6 (90.4–92.7)	89.4 (86.4–91.9)	90.6 (88.0–92.7)	91.3 (83.2–95.7)	93.6 (89.7–96.2)	86.1 (75.1–92.7)	91.0 (87.1–93.8)	92.1 (91.0–93.2)	89.0 (86.7–90.0)^††^	93.1 (91.8–94.2)	90.1 (88.4–91.5)^††^	91.7 (86.0–95.3)	77.3 (69.5–83.5)^††^
**Hib**													
Primary series**^¶¶^**	93.8 (92.9–94.6)	90.2 (86.8–92.8)^††^	92.3 (89.6–94.3)	92.6 (85.9–96.3)	95.0 (91.4–97.2)	91.4 (82.6–96.0)	91.0 (86.8–93.9)	94.0 (92.9–94.9)	90.5 (88.4–92.2)^††^	95.4 (94.4–96.1)	91.2 (89.5–92.7)^††^	92.9 (86.8–96.3)	78.1 (70.1–84.3)^††^
Full series**^¶¶^**	83.0 (81.7–84.4)	75.6 (71.6–79.2)^††^	82.1 (78.6–85.1)	82.9 (74.9–88.8)	83.5 (77.2–88.4)	—***	83.0 (78.6–86.7)	83.6 (82.1–85.0)	77.4 (74.8–79.9)^††^	85.5 (83.7–87.1)	79.6 (77.5–81.5)^††^	82.6 (76.2–87.5)	61.5 (52.7–69.6)^††^
**HepB**													
≥3 doses	91.3 (90.0–92.4)	90.0 (86.9–92.5)	89.1 (85.7–91.8)	91.0 (83.4–95.3)	93.8 (89.9–96.3)	86.0 (75.0–92.6)	88.8 (83.9–92.4)	90.5 (89.0–91.8)	90.5 (88.3–92.4)	91.2 (89.6–92.6)	90.4 (88.6–92.0)	92.4 (86.3–95.9)	78.8 (71.1–84.9)^††^
Birth dose^†††^	68.6 (66.7–70.4)	74.0 (69.9–77.8)^††^	73.4 (68.9–77.4)^††^	75.0 (64.6–83.1)	73.8 (66.1–80.2)	—***	70.7 (64.3–76.3)	70.1 (68.1–72.0)	74.9 (71.8–77.7)^††^	68.0 (65.8–70.2)	74.3 (71.6–76.8)^††^	73.7 (67.4–79.2)	63.9 (55.1–71.8)
**Varicella^§§^** (≥1 dose)	90.8 (89.6–91.9)	89.9 (86.9–92.3)	90.2 (87.4–92.4)	90.9 (82.9–95.4)	94.2 (90.2–96.6)^††^	86.7 (75.8–93.1)	89.3 (85.0–92.5)	91.2 (89.9–92.3)	89.3 (87.3–91.0)	92.3 (91.0–93.5)	89.8 (88.2–91.3)^††^	91.0 (85.2–94.7)	75.9 (68.1–82.3)^††^
**PCV**													
≥3 doses	93.1 (92.1–94.0)	88.3 (84.9–91.1)^††^	92.2 (89.5–94.3)	92.2 (85.5–95.9)	89.8 (83.7–93.8)	89.1 (79.0–94.7)	90.7 (86.4–93.7)	93.0 (91.8–94.1)	89.9 (87.8–91.7)^††^	94.0 (92.8–95.1)	90.4 (88.7–91.9)^††^	93.8 (87.9–96.9)	79.2 (71.5–85.2)^††^
≥4 doses	84.1 (82.6–85.5)	74.5 (70.0–78.5)^††^	81.4 (77.9–84.4)	80.1 (71.3–86.7)	81.0 (72.9–87.1)	82.9 (71.9–90.2)	82.9 (78.4–86.6)	84.2 (82.6–85.8)	76.8 (74.1–79.4)^††^	86.9 (85.1–88.5)	78.4 (76.2–80.5)^††^	79.2 (69.2–86.5)	62.0 (53.2–70.0)^††^
**HepA** (≥2 doses)	60.0 (58.1–61.9)	53.9 (49.6–58.1)^††^	63.6 (59.7–67.2)	69.8 (60.1–78.0)^††^	69.7 (63.6–75.3)^††^	—***	57.4 (51.7–62.9)	61.9 (60.0–63.8)	56.9 (53.9–59.9)^††^	62.7 (60.6–64.8)	60.0 (57.5–62.4)	59.1 (50.4–67.3)	42.6 (33.5–52.3)^††^
**Rotavirus^§§§^**	77.3 (75.6–78.8)	67.2 (62.9–71.3)^††^	73.0 (69.0–76.5)^††^	—***	71.8 (63.6–78.7)	—***	73.4 (68.2–78.1)	78.2 (76.4–79.9)	65.5 (62.4–68.5)^††^	80.7 (78.7–82.6)	68.7 (66.3–71.0)^††^	72.8 (63.4–80.5)	59.9 (51.2–68.0)^††^
**Combined series^¶¶¶^**	72.2 (70.4–73.9)	64.1 (59.6–68.3)^††^	71.0 (67.1–74.6)	68.5 (58.2–77.2)	72.3 (64.6–78.9)	—***	71.5 (66.1–76.3)	72.5 (70.7–74.3)	66.0 (63.0–68.9)^††^	74.9 (72.8–76.9)	68.1 (65.7–70.3)^††^	69.5 (60.4–77.3)	51.0 (41.9–60.0)^††^

**Abbreviations:** CI = confidence interval; DTaP = diphtheria and tetanus toxoids and acellular pertussis vaccine; HepA = hepatitis A vaccine; HepB = hepatitis B vaccine; Hib = *Haemophilus influenzae* type b vaccine; MMR = measles, mumps, and rubella vaccine; PCV = pneumococcal conjugate vaccine.

* Children's race/ethnicity was reported by parent or guardian. Children identified in this report as white, black, Asian, American Indian/Alaska Native, Native Hawaiian or other Pacific Islander, or multiple races were reported by the parent or guardian as non-Hispanic. Children identified as being of multiple races had more than one race category selected. Children identified as Hispanic might be of any race.

^†^ Children were classified as below poverty if their total family income was less than the poverty threshold specified for the applicable family size and number of children aged <18 years. Children with total family income at or above the poverty threshold specified for the applicable family size and number of children aged <18 years were classified as at or above poverty. A total of 560 children with adequate provider data and missing data on income were excluded from the analysis. Poverty level was based on 2015 U.S. Census poverty thresholds (https://www.census.gov/data/tables/time-series/demo/income-poverty/historical-poverty-thresholds.html).

^§^ Children’s health insurance status was reported by parent or guardian. “Other insurance” includes the Children’s Health Insurance Program (CHIP), military insurance, Indian Health Service, and any other type of health insurance not mentioned elsewhere.

^¶^ Children in the 2016 National Immunization Survey-Child were born January 2013–May 2015.

** Includes children who might have been vaccinated with diphtheria and tetanus toxoids, or diphtheria and tetanus toxoids, and pertussis vaccine.

^††^ Statistically significant (p<0.05) difference from the referent group.

^§§^ Includes children who may have been vaccinated with measles, mumps, rubella, and varicella vaccine.

^¶¶^ Hib primary series: receipt of ≥2 or ≥3 doses, depending on product type received; full series: primary series and booster dose includes receipt of ≥3 or ≥4 doses, depending on product type received.

*** Estimate not available because the 95% CI was ≥20.

^†††^ One dose HepB administered from birth through age 3 days.

^§§§^ Includes ≥2 or ≥3 doses, depending on product type of vaccine received (≥2 doses for Rotarix [RV1], or ≥3 doses for RotaTeq [RV5]).

^¶¶¶^ The combined seven vaccine series (4:3:1:3*:3:1:4) includes ≥4 doses of DTaP, ≥3 doses of poliovirus vaccine, ≥1 dose of measles-containing vaccine, the full series of Hib (≥3 or ≥4 doses, depending on product type of vaccine), ≥3 doses of HepB, ≥1 dose of varicella vaccine, and ≥4 doses of PCV.

For most vaccines, coverage among children living below the federal poverty level was lower than coverage among those living at or above the federal poverty level (Table 2). The largest gaps were for rotavirus vaccine (12.7 percentage points), ≥4 PCV doses (7.4 percentage points), the 7-vaccine series (6.5 percentage points), and the full series of Hib (6.2 percentage points). HepB birth dose coverage was higher among children living below the poverty level.

Vaccination coverage varied widely by health insurance status, with highest coverage (other than for the HepB birth dose) among children with private insurance, and lowest among uninsured children (Table 2). Compared with children who had private insurance, percentage point differences for children insured by Medicaid ranged from -2.5 for ≥3 doses of poliovirus vaccine and ≥1 dose of varicella to -12.0 for rotavirus vaccination, and for uninsured children, ranged from -12.4 for ≥3 doses of HepB to -24.9 for ≥4 doses of PCV. A higher percentage of uninsured children had received no vaccinations (3.4%) compared with those insured by Medicaid (0.8%) or private insurance (0.6%).

## Trends in Vaccination Coverage

Coverage in 2016 was statistically significantly lower than in 2015 by 1.3 to 2.3 percentage points for ≥3 doses of DTaP, ≥3 doses of poliovirus vaccine, the primary series of Hib, ≥3 doses of HepB, and ≥3 and ≥4 doses of PCV (Table 1). Analysis of trends in coverage by age 2 years, by month and year of birth (*3*), indicated that coverage among children included in combined data from the 2015 and 2016 NIS-Child (born January 2012 through January 2015) did not change for any vaccination. When expanded to children included in the 2012–2016 NIS-Child (children born January 2009 through January 2015), coverage did not change for ≥4 doses of DTaP, ≥1 dose of MMR, the full series of Hib, ≥1 dose of varicella, and ≥4 doses of PCV (Figure). Coverage over 12 consecutive birth months declined by 0.3 percentage points for ≥3 doses of poliovirus vaccine and increased for ≥3 doses of HepB (0.6 percentage points) and ≥2 doses of HepA (1.7 percentage points). Rotavirus vaccination coverage by age 19 months also increased by 1.4 percentage points per 12 birth months. No differences in 2015 and 2016 survey respondent characteristics, changes in survey operations, or errors in processing of survey data were identified.

**FIGURE:**
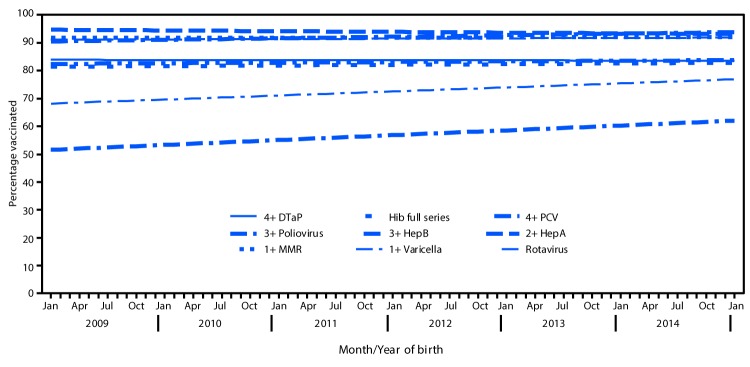
Estimated linear trend in coverage with selected vaccines* by age 24 months,^†^ by month and year of birth^§^ — National Immunization Survey-Child, United States, 2012–2016 **Abbreviations**: DTaP = diphtheria and tetanus toxoids and acellular pertussis vaccine; HepA = hepatitis A vaccine; HepB = hepatitis B vaccine; Hib = *Haemophilus influenzae* type b vaccine; MMR = measles, mumps, and rubella vaccine; PCV = pneumococcal conjugate vaccine. * Hib full series: receipt of ≥3 or ≥4 doses, depending on product type received (primary series and booster dose). Rotavirus includes ≥2 or ≥3 doses, depending on product type of vaccine received (≥2 doses for Rotarix [RV1], or ≥3 doses for RotaTeq [RV5]). ^†^ Except for rotavirus, vaccination coverage was assessed before the child reached age 24 months. The Kaplan-Meier method was used to account for censoring of vaccination status for children assessed before age 24 months. Rotavirus vaccination was assessed before the child reached age 19 months and might include some vaccinations reported as received after the maximum recommended age of 8 months, zero days. ^§^ Estimated linear relationship between month and year of birth and vaccination coverage, based on weighted linear regression analysis using the inverse of the estimated variance of each point estimate to construct the weights. Estimated percentage point change over 12 consecutive birth months: 4+ DTaP -0.05 (-0.4, 0.3); 3+ Poliovirus -0.3 (-0.5, -0.006); 1+ MMR 0.05 (-0.2, 0.3); Hib full series 0.2 (-0.1, 0.6); 3+ HepB 0.6 (0.3, 0.9); 1+ Varicella 0.1 (-0.1, 0.4); 4+ PCV 0.2 (-0.1, 0.6); 2+ HepA 1.7 (1.2, 2.3); Rotavirus 1.4 (1.0, 1.9).

## Discussion

Coverage with recommended vaccines for children aged 19–35 months continues to be high and stable but remains below 90% for vaccines that require booster doses during the second year of life (≥4 doses of DTaP and PCV as well as Hib full series) and for other recommended vaccines (HepB birth dose, rotavirus, and HepA). Disparities in coverage persisted for black children and those living below the poverty level, and coverage was generally lower for children who were uninsured or covered by Medicaid than among those with private insurance. These disparities indicate that improvements are needed in access to and delivery of age-appropriate immunization to all children, regardless of insurance or financial status (i.e., “the immunization safety net”).

Health insurance and poverty status are interrelated factors associated with lower vaccination coverage in young children. Compared with children who had only private insurance, those with Medicaid had lower coverage, and those who were uninsured had much lower coverage, for most vaccines. Uninsured children, who account for 3.0% of the 2016 NIS-Child weighted sample, are eligible for the VFC program, which was designed to increase access to vaccination among children through age 18 years who might not otherwise be vaccinated because of inability to pay. Some families might not be aware of the VFC program, be unable to afford fees associated with visits to a vaccine provider, or might need assistance locating a physician who participates in the VFC program. Children living below poverty and up to a certain percentage above the poverty level are eligible for Medicaid (42.5% of 2016 NIS-Child population met the minimum Medicaid eligibility level of 138%) and are entitled to VFC vaccines. Barriers to health care access and use among the publicly insured include language barriers, lack of trust in providers, transportation problems, inconvenient office hours, and other provider- and system-level factors (*4*). Medicaid patients also tend to experience more breaks in insurance coverage than do privately insured children, and discontinuities in insurance coverage have been associated with lower vaccination coverage (*5*). NIS-Child establishes insurance status at the time of interview, not necessarily at the time of recommended vaccination, with enrollment in insurance sometimes occurring after the time window for receipt of certain vaccines. CDC has undertaken a number of activities designed to elucidate potential barriers to early childhood vaccination from the perspective of the state immunization programs and health care providers enrolled in the VFC program. There are also plans to assess parental experience with and barriers to accessing vaccination services.

Lower vaccination coverage among black children than among white children has been explained by differences in poverty status in past years (*6*), but in 2016, racial disparities were found among children living at or above the poverty level for some vaccines. This might reflect incomplete control for poverty status, because black children living above poverty could still live in lower income households, on average, than do white children. In the 2016 NIS-Child, the proportion of white children living in households with an income to poverty ratio of ≥4 was twice that of black children.

During routine checks for accuracy of the 2016 NIS-Child data, statistically significant differences were observed in vaccination coverage by age 19 months estimated from the 2016 compared with the 2015 surveys, among birth cohorts eligible to be included in both survey years (*3**,**7*). These differences were observed for 9 of the 15 vaccine doses evaluated and might indicate a systematic change in bias of the survey from 2015 to 2016. However, no differences were found in survey respondent characteristics and survey operations, and no errors were identified in processing of survey data; thus, it is possible that these differences might be attributable to random variation.

The observed vaccination coverage differences among birth cohorts eligible for both survey years contributed to drops in annual estimates of vaccination coverage using the entire sample of survey respondents from 2015 to 2016, but do not provide evidence for change in vaccination coverage over time (*3*). When trends were assessed more directly by month and year of birth from January 2012 through January 2015 (*3*), coverage by age 2 years was stable for all vaccines. When trends were assessed over a longer range of births, from January 2009 through January 2015, coverage was stable for most vaccines; for other vaccines, estimated change over twelve monthly birth cohorts was within one percentage point, and increased by 1–2 percentage points for rotavirus vaccination and ≥2 doses of HepA. Further evaluations of methods for assessing trends in survey accuracy and vaccination coverage using NIS-Child data are needed. Improved data quality of immunization information systems (IIS) will facilitate their use as another data source for population-based coverage assessment (*8*).

The findings in this report are subject to at least three limitations that have been previously described, including exclusion of households without telephones, nonresponse bias, and incomplete vaccination histories reported by providers (*6*). Total survey error has been evaluated in a sensitivity analysis accounting for these errors. Analyses of 2012 and 2013 data revealed that NIS-Child might have underestimated true vaccination coverage in those years by ≤4 percentage points for MMR, ≤5 percentage points for ≥4 doses of DTaP, and 5 percentage points for a 6-vaccine series that excluded PCV (*9*,*10*). Changes in annual vaccination coverage estimates should be interpreted with caution (*3*), particularly when they are smaller than the survey margin of error.

These data indicate that the immunization safety net is not reaching all children early in life. Coverage could be increased with implementation of evidence-based interventions, such as provider reminders to eliminate missed opportunities to vaccinate, standing orders to provide vaccination whenever appropriate, and use of IIS to track vaccination administration.*** In addition to maintaining the strong U.S. immunization program, innovative approaches are needed to identify children not reached by the current safety net, including using local level IIS data. Continued vaccination coverage assessment using the NIS-Child will guide efforts to improve vaccination coverage. Data completeness and functionality of IIS have improved in recent years (*8*); however, additional progress is needed to maximize IIS utility for vaccination coverage assessments at state and local levels.

**Summary**

**What is already known about this topic?**

Vaccination is an effective method for reducing the impact of many diseases among young children in the United States. For over 20 years, the National Immunization Survey-Child has gathered data on children aged 19–35 months to assess coverage with the vaccines recommended by the Advisory Committee on Immunization Practices (ACIP).

**What is added by this report?**

Coverage with most recommended vaccines remained stable and high in 2016; coverage was ≥90% for polio, measles, mumps, and rubella, varicella, and hepatitis B vaccines, and lowest (61%–74%) for hepatitis A, the birth dose of hepatitis B, and rotavirus vaccines, and the combined 7-vaccine series. For most vaccines, coverage was lower among black children, children living below the federal poverty level, and children who were uninsured or covered by Medicaid compared with white children, children living at or above the federal poverty level, and children with private insurance.

**What are the implications for public health practice?**

Continued collaboration between CDC and state immunization programs to further elucidate and address disparities in coverage by poverty status should provide valuable information while strategies needed for improving access to and delivery of age-appropriate immunization are identified. Health care providers can increase vaccination coverage using evidence-based strategies such as provider reminders, standing orders to provide vaccination whenever appropriate, and immunization information systems.

## Footnotes

* Child’s race/ethnicity was reported by his/her parent or guardian. Children categorized in this report as white, black, American Indian/Alaska native, Asian, native Hawaiian/other Pacific Islander, or multiple races were identified as non-Hispanic by the parent or guardian. Children identified as being of multiple races had more than one race category designated. Children identified as Hispanic might be of any race.

† Poverty level uses income and family size to categorize households into those 1) at or above the poverty level, and 2) below the poverty level. Poverty level was based on 2015 U.S. Census poverty thresholds (https://www.census.gov/data/tables/time-series/demo/income-poverty/historical-poverty-thresholds.html).

§ https://www.cdc.gov/vaccines/programs/vfc/index.html.

¶ Estimates for states, selected local areas, and territories are available online at https://www.cdc.gov/vaccines/imz-managers/coverage/childvaxview/data-reports/index.html. The local areas sampled separately for the 2016 NIS-Child included areas that receive federal Section 317 immunization funds and are included in the NIS-Child sample every year (Chicago, Illinois; New York, New York; Philadelphia County, Pennsylvania; Bexar County, Texas; and Houston, Texas) and two additional sample areas (El Paso County, Texas and Dallas County, Texas). The 2016 NIS-Child was also conducted in Guam, Puerto Rico, and the U.S. Virgin Islands. This report includes national estimates, excluding the territories.

** Further details regarding the statistical methodology of NIS-Child are available in the NIS-Child Data User’s Guide 2015, which is available at https://www.cdc.gov/vaccines/imz-managers/nis/datasets.html.

†† The Council of American Survey Research Organizations (CASRO) household response rate is calculated as the product of the resolution rate (percentage of the total telephone numbers called that were classified as nonworking, nonresidential, or residential), screening completion rate (percentage of known households that were successfully screened for the presence of age-eligible children), and the interview completion rate (percentage of households with one or more age-eligible children that completed the household survey). The CASRO household response rate is equivalent to the American Association for Public Opinion Research type 3 response rate http://www.aapor.org/AAPOR_Main/media/publications/Standard-Definitions20169theditionfinal.pdf.

§§ Children with receipt of at least one vaccination reported by a provider and those who received no vaccinations were considered to have adequate provider data.

¶¶ The combined 7-vaccine series (4:3:1:3*:3:1:4) includes ≥4 doses of DTaP; ≥3 doses of poliovirus vaccine; ≥1 dose of measles-containing vaccine; ≥3 or ≥4 doses of Hib (depending upon product type of vaccine); ≥3 doses of HepB; ≥1 dose of varicella vaccine; and ≥4 doses of PCV.

*** https://www.thecommunityguide.org/topic/vaccination.
